# Effects of Passive Leg Raising And Volume Expansion On Mean Systemic Pressure And Venous Return in Shock in Humans

**DOI:** 10.1186/2197-425X-3-S1-A16

**Published:** 2015-10-01

**Authors:** L Guérin, JL Teboul, R Persichini, M Dres, C Richard, X Monnet

**Affiliations:** AP-HP, Hôpitaux Universitaires Paris-Sud, Hôpital de Bicêtre 2: Univ Paris-Sud, Faculté de Médecine Paris-Sud, EA 4533, Intensive Care Unit, Le Kremlin Bicêtre, France

## Introduction

The passive leg raising (PLR) test has been developed for predicting fluid responsiveness. Nevertheless, one could question its ability to induce a significant increase in the stressed blood volume. Also, the fact that some patients do not respond to volume expansion by augmenting cardiac output could theoretically be related to the absence of preload dependence, but also to an insufficient increase in stressed blood volume.

## Objectives

To assess the effects of increases in preload induced by passive leg raising (PLR) and fluid infusion on the pressure gradient between mean systemic pressure (Psm) and central venous pressure (CVP) and on the resistance to venous return (Rvr).

## Methods

In 30 patients with an acute circulatory failure, in order to estimate the venous return curve, we constructed the regression line between pairs of cardiac index (CI) and CVP. Values were measured during end-inspiratory and end-expiratory ventilatory occlusions performed at two levels of positive end-expiratory pressure. The x-axis intercept was used to estimate Psm and the inverse of the slope to quantify Rvr. These measurements were obtained at baseline, during PLR and after fluid infusion.

## Results

In volume-responders (n = 15), Psm increased from 25 ± 13 to 31 ± 13 mmHg during PLR and to 32 ± 17 mmHg after fluid infusion. CVP increased from 7 ± 3 mmHg to 9 ± 4 mmHg during PLR and to 9 ± 4 mmHg after fluid infusion. Thus, in these patients, the Psm-CVP gradient increased significantly during PLR and fluid infusion. By contrast in non-volume responders, PLR and fluid infusion increased Psm and CVP to a similar extent, so that the Psm-CVP gradient was unchanged. Rvr was modified neither by PLR nor by fluid infusion in both groups.

## Conclusions

In preload-dependent patients, PLR and fluid infusion increased venous return owing to an increase in Psm higher than in CVP, with unchanged Rvr. in preload-independent patients, neither PLR nor fluid infusion changed venous return, Psm-CVP gradient and Rvr.

